# Redundant laboratory testing on referral from general practice to the outpatient clinic: a post-hoc analysis

**DOI:** 10.3399/BJGPO.2021.0134

**Published:** 2021-12-08

**Authors:** Bram EL Vrijsen, Maarten J ten Berg, Wouter W van Solinge, Jan Westerink

**Affiliations:** 1 Department of Internal Medicine, Division Internal Medicine and Dermatology, University Medical Center Utrecht, Utrecht University, Utrecht, The Netherlands; 2 Central Diagnostic Laboratory, Division Laboratories, Pharmacy and Biomedical Genetics, University Medical Center Utrecht, Utrecht University, Utrecht, The Netherlands

**Keywords:** clinical laboratory techniques, clinical laboratory services, general practice, hospitals, laboratories, medical overuse, outpatient clinics, hospital, referral and consultation

## Abstract

**Background:**

Inappropriately repeated laboratory testing is a commonly occurring problem. However, this has not been studied extensively in the outpatient clinic after referral by GPs.

**Aim:**

The aim of this study was to investigate how often laboratory tests ordered by the GP were repeated on referral to the outpatient clinic, and how many of the normal test results remained normal on repetition.

**Design & setting:**

This is a post-hoc analysis of a study on laboratory testing strategies in patients newly referred to the outpatient clinic between April 2015 and April 2017.

**Method:**

All patients who had a referral letter including laboratory test results ordered by the GP were included. These results were compared with the laboratory test results ordered in the outpatient clinic.

**Results:**

Data were available for 295 patients, 191 of which had post-visit testing done. In this group, 56% of tests ordered by the GP were repeated. Tests with abnormal results were repeated more frequently than tests with normal results (65% versus 53%; *P*<0.001). A longer test interval was associated with slightly smaller odds of tests being repeated (odds ratio [OR] 0.97, 95% confidence interval [CI] = 0.95 to 0.99; *P* = 0.003). Of the tests with normal test results that were repeated, 90% remained normal. This was independent of testing interval or testing strategy.

**Conclusion:**

Laboratory tests ordered by the GP are commonly repeated on referral to the outpatient clinic. The number of test results remaining normal on repetition suggests a high level of redundancy in laboratory test repetition.

## How this fits in

Laboratory tests are frequently repeated when patients are transferred from one healthcare provider to another. This study shows that repeat testing is also common on referral from general practice to the outpatient clinic. Furthermore, it was found that in the majority of cases, these repetitions yield no new information, suggesting a high level of redundancy.

## Introduction

When patients are referred from general practice to the outpatient clinic, history taking and physical examination are typically repeated by the physician in the hospital uncontroversially. However, repeating imaging or laboratory testing is generally deemed to be wasteful in many cases.^1^ Inappropriate duplicate testing is a frequently occurring example of overutilisation, with an estimated 21% of overall laboratory tests being unwarranted.^2^ Ordering unnecessary tests not only leads to increased healthcare costs, but can also compromise the diagnostic process by leading to more false positive results.^3^ Furthermore, overtesting places a burden on patients, by subjecting them to more phlebotomies and potential follow-up testing, which in turn can lead to anxiety about test results.^4,5^

One of the causes of inappropriate repeat testing is unawareness that the test has already been ordered.^6^ This can occur when the care for patients is being transferred from one healthcare provider to another. For example, in transfers between emergency departments, duplication rates of 32%–88% have been found.^7–11^

Repetition rates for patients being transferred from general practice to the hospital have been studied less extensively. One study, investigating repetition rates for patients who were admitted to the medical department of a hospital, found that 63% of tests ordered by the GPs in the previous year were repeated on admission.^12^

For patients referred to the outpatient clinic, there are only data on repetition rates in patients referred for second opinions, where up to 90% of laboratory tests were repetitions of tests ordered by the original physician.^13,14^ Yet no data are available on patients newly referred to the outpatient clinic by their GP. Given the paucity of the data, a post-hoc analysis was performed of a previously published study by the authors on laboratory test strategies in the outpatient clinic.^15^

The aim of the current study was to determine how often laboratory tests ordered by GPs are repeated in the outpatient clinic after referral, depending on the test, the test result, the test interval, and the laboratory test strategy at the outpatient clinic. Second, to discover how often tests with normal results remain normal on repetition, depending on the test, the test interval, and the laboratory test strategy at the outpatient clinic.

## Method

### Setting and patient selection

This study is a post-hoc analysis of a study investigating the effect of pre-visit laboratory testing on the time to diagnosis in outpatients.^15^ In this previous study by the authors, patients newly referred to the internal medicine outpatient clinic of the University Medical Centre Utrecht (UMC Utrecht), a tertiary hospital in the Netherlands, from April 2015 to April 2017, were allocated to either pre-visit or post-visit laboratory testing.

In the pre-visit arm, patients had a standardised panel of laboratory tests done 1 hour before the first visit to the outpatient clinic, so the test results were available to the treating physician during the visit. In the post-visit arm, laboratory testing was done after the first visit at the discretion of the treating physician.

For the current study, all subjects were included for whom a referral letter from their GP was available that included laboratory test results. From these referral letters all results from laboratory tests, including test dates, were collected by one of the authors. Data on the laboratory testing done in the outpatient clinic were already available from the previous study.

### Measures

All test results were classified as being either normal (that is, within the reference range) or abnormal (that is, outside of the reference range). Because the referral letters generally did not include reference intervals or information on which laboratory had performed the tests, the reference intervals could not be obtained. For this reason, the reference intervals of the laboratory of the UMC Utrecht were used.

Both leucocyte differentiation and urine screening, which comprise several different tests, were considered as one test each. Results were considered to be abnormal if ≥1 of the constituent tests were outside the reference range.

In several cases the reference range was adjusted, as for some tests a value below or above the reference range is generally clinically irrelevant. The lower limits of normal were removed for the activated partial thromboplastin time , C-reactive protein, creatinine, erythrocyte sedimentation rate (ESR), haemoglobin A1c, international normalised ratio, prothrombin time, and urea. The upper limits of normal were removed for vitamins B12 and D.

For every test ordered by the GP, the test interval was calculated as the time in weeks between the test ordered by the GP and the date of the first visit to the outpatient clinic.

The laboratory strategy (either pre-visit testing or post-visit testing) was recorded 'as treated', meaning that all patients were assigned to the laboratory strategy they underwent in the original study, rather than the strategy to which they were originally allocated.

### Outcomes

The primary outcome was the number of tests ordered by the GP that were repeated in the outpatient clinic. This was only studied in the patients who had undergone post-visit testing (because the standardised panels used in the pre-visit testing group inevitably lead to repeat testing, including the group would lead to inflated repetition rates). The secondary outcome was the number of repeated tests remaining normal on repetition. This was studied in all patients.

### Statistical analysis

The difference in repetition rates for tests with normal versus abnormal results was statistically tested using the χ^2^ test. ORs for tests being repeated, and for normal tests remaining normal on repetition depending on the time interval, were calculated using a logistic mixed-effects model, with the individual patients as random effect. The number of repeated tests and repeats of normal tests per patient were compared between laboratory testing strategies using the Wilcoxon rank-sum test. Repetition rates and rates of normal tests remaining normal were also calculated for the 10 most commonly ordered tests. All analyses were performed in R (version 4.0.3).

## Results

### Baseline characteristics

Of the 594 eligible subjects in the original study, referral letters could be retrieved in 449 cases (76%), 295 of which (66%) included laboratory test results. All 295 subjects were included in this study. The included patients visited the internal medicine outpatient clinic between April 2016 and November 2017. The mean age was 52.7 years (95% CI = 50.5 to 54.8 years) and 66% were female. Pre-visit testing was done in 104 patients and post-visit testing in 191. Baseline characteristics of these patients are reported in Table 1.

**Table 1.** Baseline characteristics

Characteristic	Pre-visit testing	Post-visit testing
Total, *n*	104	191
Age, years (95% CI)	53.4 (49.7 to 57.1)	52.3 (49.6 to 54.9)
Female*, n* (%)	66 (63)	129 (68)
**Referral reason, *n* (%)^a^**		
Abdominal complaints	12 (12)	23 (12)
Abnormal laboratory test	16 (15)	28 (15)
Anaemia	12 (12)	28 (15)
Fatigue	37 (36)	47 (25)
Lymphadenopathy/suspected malignancy	1 (1)	8 (4)
Weight loss	15 (14)	29 (15)
Other	24 (23)	43 (23)

^a^Categories are not exclusive.

### Rate of repeated tests

In the post-visit testing group, the median number of laboratory tests ordered by the GP was 14.0 (interquartile range [IQR] 10.0–18.0) per patient, of which 8.0 (IQR 4.0–11.0) were repeated in the outpatient clinic. Overall, 1440 out of 2587 tests (56%) were repeated. Median time interval was 32 days (IQR 19–67 days). Tests with abnormal results were repeated more frequently than those with normal results (*n* = 337/516, 65% versus *n* = 1103/2071, 53%; *P*<0.001). A longer test interval was associated with slightly smaller odds of tests being repeated (OR 0.97, 95% CI = 0.95 to 0.99; *P* = 0.004).

The number of repeated tests per patient for the two different laboratory testing strategies is reported in Table 2. Pre-visit testing led to more repetitions (median 9.5, IQR 6.0–13.0) compared with post-visit testing (median 8.0, IQR 4.0–11.0; *P*<0.001).

**Table 2.** Number of repeats of primary care physician’s tests depending on laboratory testing strategy in the outpatient clinic

Tests, normal results, and repeat tests, median (IQR)	Pre-visittesting (*n* = 104)	Post-visittesting (*n* = 191)	*P* value
Tests	12.5 (8.8–17.0)	14.0 (10.0–18.0)	—
Normal results	9.5 (6.0–14.0)	11.0 (8.0–14.0)	—
Repeats overall	9.5 (6.0–13.0)	8.0 (4.0–11.0)	*P*<0.001^a^
Repeats of normal results	8.0 (3.8–10.0)	5.0 (3.0–9.0)	*P*<0.001^a^

^a^Statistically tested using the Wilcoxon rank-sum test. IQR = interquartile range.

The 10 tests most commonly ordered by the GPs are listed in Table 3. At the top of this list are haemoglobin, mean corpuscular volume (MCV), and white blood cell count. With the exception of glucose, the tests most commonly ordered by the GPs are also among the most commonly repeated tests, with common haematology parameters and creatinine being repeated in >85% of cases.

**Table 3.** Ten most commonly ordered tests with repetition rates and rates of normal tests remaining normal on retesting

Test	Tests by primary care physician, *n*	Repeats, *n* (%)	Normal results on first test being repeated, *n* (%)	Normal repeats remaining normal, *n* (%)
Haemoglobin	247	217 (88)	141 (84)	134 (95)
MCV	237	204 (86)	170 (85)	163 (96)
Leucocytes	227	198 (87)	154 (85)	136 (88)
Creatinine	213	185 (87)	152 (85)	147 (97)
ESR	198	151 (76)	96 (77)	95 (99)
TSH	192	141 (73)	129 (75)	125 (97)
Glucose	191	114 (60)	73 (57)	57 (78)
Thrombocytes	188	162 (86)	142 (85)	136 (96)
ALT	164	136 (83)	111 (80)	103 (93)
Leucocyte differential	156	114 (73)	106 (72)	82 (77)

ALT = alanine transaminase. ESR = erythrocyte sedimentation rate. MCV = mean corpuscular volume. TSH = thyroid stimulating hormone.

### Rate of repeated tests remaining normal

Of all tests with normal results that were repeated in both the pre-visit and post-visit testing groups, 1678 (90%) remained normal on repetition. This was independent of the testing interval (OR 0.99, 95% CI = 0.97 to 1.01; *P* = 0.1) or testing strategy (OR 1.06, 95% CI = 0.73 to 1.53; *P* = 0.76).

The rates of normal test results remaining normal on repetition were even higher for the most commonly ordered tests: >95% of the repeated tests for normal results of haemoglobin, MCV, creatinine, ESR, thyroid stimulating hormone (TSH), and thrombocyte count remained normal (Table 3).

The tests with the highest rates of normal test results becoming abnormal on repetition are shown in Table 4. Urine screening was the only test in which normal test results became abnormal on repetition in the majority of cases.

**Table 4.** Tests with the highest rates of normal test results becoming abnormal on retesting

Test	Normal results being repeated, *n* (% of normal results)	Normal repeats remaining normal, *n* (% of normal repeats)
Urine screening	14 (82)	4 (29)
Creatine kinase	2 (22)	1 (50)
Iron	3 (14)	2 (67)
Cholesterol	4 (6)	3 (75)
Reticulocytes	8 (29)	6 (75)
Leucocyte differential	106 (72)	82 (77)
Potassium	90 (74)	70 (78)
Glucose	73 (57)	57 (78)
Sodium	83 (75)	67 (81)
Ferritin	11 (31)	9 (82)

## Discussion

### Summary

This study shows that repeated ordering of tests is common on referral from general practice, with 56% of tests ordered by the GP being repeated in the outpatient clinic.

To the authors’ knowledge, this study is the first to specifically investigate the rate of repeating laboratory tests on referral from the GP to the outpatient clinic.

It was found that tests with normal results were repeated only slightly less frequently than tests with abnormal results. In the majority of cases, tests with normal results remained normal on repetition, which suggests a high level of redundancy in laboratory test repetition. This may be especially true for tests such as the ESR, TSH, or creatinine, which are known to be generally stable over time, and almost never became abnormal on repetition in this study.

### Strengths and limitations

One strength of this study is that it includes an unselected group of patients with a wide variety of referral reasons, which increases the external validity of the study (the original study^15^ included all newly referred patients to the outpatient clinic).

This study also has several limitations. First, there was no information available to determine why the treating physicians at the outpatient clinic decided to repeat some tests. Consequently, the appropriateness of these repetitions using retrospective data could not be quantified. Second, this study was conducted in a single tertiary centre, so the results may not be applicable to other settings. Finally, the hospital’s reference ranges were also used for the tests ordered by the primary care physicians in other laboratories, because those laboratories’ reference ranges were not available. As a result, several laboratory tests may have been misclassified as either normal or abnormal.

### Comparison with existing literature

The repetition rate found in this study is significantly higher than in a previous Dutch study, which, when studying data from patients who had laboratory testing done both at the primary care physician and at the hospital, found only 0.5% of laboratory tests to be duplicates.^16^ In that study, however, the number of duplicate tests was compared with the total number of tests performed at the hospital’s laboratory, not just the tests performed on newly referred patients. Furthermore, it only considered tests to be duplicates if repeated within a 7-day time period.

The results of the present study can also be compared with data from studies investigating a similar question but in a different setting. For example, one study focused on repetitions of laboratory tests ordered by the primary care physician for patients on admission to the hospital.^12^ In this study, 300 consecutive patients who had been admitted to the medical department of a general hospital were included. Their primary care physicians were asked to provide information on laboratory testing performed in the previous year. Data were available for 202 patients, and showed that 63% of the tests ordered by the primary care physician in the previous year were repeated on admission. However, as these patients were admitted to the hospital, there was an a priori higher chance of the results from the repeated test being abnormal. This underlines the notion that repetition of a laboratory test does not necessarily mean redundancy, as the patient’s condition might change rapidly.

The definition of whether or not a repeated test is redundant is hampered by the absence of good data and guidelines, as well as by reasons related to the psychology of both the patient and the doctor. In many cases, therefore, tests may not be entirely appropriate or inappropriate, but rather fall into a grey zone.^17^

Appropriateness of repeated laboratory test is thus often simplified. For example, one study simply defined repeat tests as appropriate if the results of the initial tests were available to the physician ordering the repeat test, on the basis that there must have been a good reason for repeating.^12^

Two other studies that have focused on repetition rates on transfer between hospitals have also investigated the appropriateness of repeat testing, using expert panels to assess each test separately. Respectively, they found 63% and 99.5% of repetitions to be inappropriate.^8,10^ No studies could be found on whether or not the physician requesting the tests thought of these retests as redundant, or what the reasons for the retesting were.

There are several possible causes of inappropriate repeat testing. The physician in the outpatient clinic may not be aware of the existing data owing to insufficient preparation. This may be because of time constraints, or it could be that physicians fail to read referral letters because they believe them to be of poor quality.^18^ Another potential reason is that the results from the first test are not available to the doctor, or are only available in an unsuitable format such as a hard copy on paper, or embedded in a referral letter that has been faxed or sent as a PDF. Furthermore, doctors might distrust the results included in the referral letter based on biased attitudes towards other laboratories. This may be compounded by the differences in reference ranges between laboratories, which make it more difficult for clinicians to correctly interpret the test result. This can also account for doctors wanting a baseline measurement in their own electronic patient file for future reference. Besides these, there could also be psychological reasons involved, including the physician’s risk aversion and the perceived expectations of the patient.^19,20^

### Implications for research and practice

The high prevalence of repeat testing, a significant proportion of which is likely to be redundant, provides an important target for improving laboratory test utilisation. By reducing the burden of venipunctures and unnecessary follow-up testing, this can lead to cost savings and improved patient care.

Possible interventions may consist of making the test results ordered by the GP more readily available to other healthcare professionals (for instance, by the coupling of different laboratory information systems) so the electronic medical records can provide clinicians with an integrated view of all laboratory tests performed either in the hospital or elsewhere.^21^ Whether this would actually reduce test repetitions warrants further study.
